# Lobar evenness of deposition/retention in rat lungs of inhaled silver nanoparticles: an approach for reducing animal use while maximizing endpoints

**DOI:** 10.1186/s12989-018-0286-9

**Published:** 2019-01-07

**Authors:** Jung Duck Park, Jin Kwon Kim, Mi Seong Jo, Young Hun Kim, Ki Soo Jeon, Ji Hyun Lee, Elaine M. Faustman, Hong Ku Lee, Kangho Ahn, Mary Gulumian, Günter Oberdörster, Il Je Yu

**Affiliations:** 1 Deparment of Preventive Medicine College of Medicine, Chung-Ang University, Seoul, South Korea; 2 Department of Nanofusion Technology, Hoseo University, Asan, South Korea; 3 HCTm CO.,LTD, Seoicheon-ro 578 beon-gil, Majang-myeon, Icheon, 17383 South Korea; 4 Department of Environmental and Occupational Health Sciences, University of Washington, Seattle, USA; 5 Department of Mechanical Engineering, Hanyang University, Ansan, South Korea; 6 National Institute for Occupational Health, University of the Witwatersrand, Johannesburg, South Africa; 7 Haematology and Molecular Medicine, University of the Witwatersrand, Johannesburg, South Africa; 8 Department of Environmental Medicine, University of Rochester, Rochester, NY USA

## Abstract

**Background:**

Information on particle deposition, retention and clearance are important for the evaluation of the risk of inhaled nanomaterials to human health. Recent revised OECD inhalation toxicity test guidelines require to evaluate the lung burden of nanomaterials after rodent subacute and subchronic inhalation exposure (OECD 412, OECD 413). These revised test guidelines require additional post-exposure observation (PEO) periods that include lung burden measurements that can inform on lung clearance behavior and translocation. The latter being particularly relevant when the testing chemical is a solid poorly soluble nanomaterial. Therefore, in the spirit of 3 R’s, we investigated whether measurement of retained lung burden of inhaled nanoparticles (NPs) in individual lung lobes is sufficient to determine retained lung burden in the total lung. If it is possible to use only one lobe, it will reduce animal use and maximize the number of endpoints evaluated.

**Results:**

To achieve these goals, rats were exposed nose-only for 1 or 5 days (6 h/day) to an aerosol of 20 nm well-dispersed silver nanoparticles (AgNPs), which is the desired particle diameter resulting in maximum deposition in the pulmonary region when inhaled as singlets. After exposure, the five lung lobes were separated and silver concentration was measured using inductively coupled plasma-mass spectrophotometer (ICP-MS). The results showed that the retention of deposited silver nanoparticle in the different lung lobes did not show any statistically significant difference among lung lobes in terms of silver mass per gram lung lobe. This novel finding of evenness of retention/deposition of inhaled 20 nm NPs in rats for all five lobes in terms of mass per unit tissue weight contrasts with earlier studies reporting greater apical lobe deposition of inhaled micro-particles in rodents. The difference is most likely due to preferred and efficient deposition of inhaled NPs by diffusion vs. additional deposition by sedimentation and impaction for micron-sized particles.

**Conclusion:**

AgNPs following acute inhalation by rats are evenly retained in each lung lobe in terms of mass per unit lung tissue weight. Accordingly, we suggest sampling any of the rat lung lobes for lung burden analysis can be used to determine deposited or retained total lung burden after short-term inhalation of NPs and using the other lobes for collecting and analyzing bronchoalveolar lavage fluid (BALF) and for histopathological analysis. Therefore, by combining lung burden measurement, histopathological tissue preparation, and BALF assay in the same rat will reduce the number of animals used and maximize the number of endpoints measured.

**Electronic supplementary material:**

The online version of this article (10.1186/s12989-018-0286-9) contains supplementary material, which is available to authorized users.

## Background

Particle size distribution of airborne particles determines which deposition mechanism, impaction, sedimentation, diffusion or interception, is most prominent under certain conditions [1]. Deposition of particles to any site of the respiratory tract may potentially induce toxicity or inflammatory reaction leading to adverse health effects. Absorption is the first step of toxicokinetics which, in addition also include distribution, metabolism, and elimination (ADME). Toxicokinetics should be considered for any substances that accumulate in the lung or translocates into and accumulates in specific extrapulmonary organs following repeated exposures or even after single exposure. Retention is a function of deposition and clearance. Toxicokinetics including deposition, retention, clearance, and translocation of nanomaterials after inhalation exposure are essential for hazard identification which is the first step of risk assessment [2] of nanomaterials. Lung burden measurements conducted in the course of repeated inhalation studies in rats may be helpful in understanding the fate and toxicity of nanomaterials. For appropriate toxicological characterization of inhaled nanomaterials, OECD inhalation toxicity test guidelines 412 (28-day subacute inhalation toxicity study) and 413 (90-day subchronic inhalation toxicity study) have been revised [3, 4]. These revisions have mandated BALF analysis for inflammatory markers such as BAL cell differential counting and LDH and total protein or albumin measurement. In addition, in particular, when the inhaled test nanomaterials are poorly soluble, the OECD guidelines recommended that lung burden should be measured to inform about the pulmonary retained dose.

Per OECD guideline, the lung burden measurements are usually performed for all test chemicals within 24 h after exposure termination and may be undertaken at one or two additional post-exposure observation (PEO) intervals [3, 4]. One or two more post-exposure observations of the lung burden measurements may, however, be required, in addition to those immediately after exposure termination, which mandates the use of more experimental animals. The original test guideline 412 (28-day subacute inhalation toxicity study) required 40 animals (5 females and 5 males per concentration group), whereas the revised test guideline requires 60 animals in order to conduct mandatory BALF analysis and lung burden measurement suggesting the use of the right lung for BALF analysis and the left lung for histopathology. Similarly, the original test guideline 413 (subchronic inhalation test) required 80 animals (10 male and 10 females per concentration group), while the revised test guideline requires 100 animals to conduct mandatory BALF analysis and lung burden measurement. Particle clearance kinetics in the lung, however, usually involves several additional post-exposure observation periods to estimate lung clearance parameters such as T_1/2_ (retention half-time), and k (clearance rate). Therefore, two additional post-exposure observation periods as suggested by OECD 412 or 413, means that the use of animal will total 120 animals for OECD 412 [3] and 160 animals for OECD 413 [4].

In the spirit of 3 R’s for ethical use of animals and given the large numbers of animals for these tests, we decided to investigate the possibility of minimizing the number of animals used but also maximizing the output of the experiments conducted. We propose that this could be achieved through designing experiments by selecting a representative lung lobe for lung burden measurement. This will be possible since rats have five lobes in their lung; right cranial lobe (RCr, or right upper lobe, or right apical lobe), right median lobe (RM, or cardiac lobe), right caudal lobe (RCa, right lower lobe, or diaphragmatic lobe), right accessory lobe (RA, right middle lobe or azygous lobe) and left lung (LL, left cranial and left caudal). We proposed it should be possible to use one representative lobe of the right lung for lung burden analysis and the other remaining lobes to be used for BALF collection following proper occlusion of the lobe. This, in turn, will help to reduce the number of animals used by conducting simultaneous measurements of the lung burden as well as the recommended BALF analysis on the same animals.

To investigate this possibility, we have analyzed particle deposition and retention in each lung lobe of the rat to identify a representative lobe for lung burden analysis. To conduct this study, AgNP having average diameter of 20 nm were generated as approximately 20–30% of inhaled particles of this size range is known to be deposited efficiently to the alveolar region and to some extent in head and tracheobronchial regions (approximately 10%) in humans – if the 20 nm particles are not agglomerated – according to International Commission on Radiological Protection (ICRP) or MPPD lung deposition models [5–8]. After 1 and 5 days of inhalation exposure to AgNP, rats were sacrificed and lung lobes were separated for lung burden analysis. Retained amounts in the lung lobes in terms of mass and particle number were evaluated. In addition, particle deposition to the lung acini where the alveoli (air sacs) are located and where major particle deposition takes place was also considered.

## Materials and methods

### Silver nanoparticle generation

Silver nanoparticles were selected for this study based on the well-established generation of dispersed 20 nm particle size described in many publications [9–11]. In addition, it is possible to study particle retention and dissolution using AgNPs. Rats were exposed to the AgNP aerosols, generated as described in previous reports [9–11], in a nose-only exposure chamber (30 ports, flow past, HCT, Icheon, Korea); 20 ports for animal exposure and 5 ports for chamber monitoring such as particle monitoring by real-time particle monitor, sampling, and chamber environmental monitoring. The generator consisted of a small ceramic heater connected to an AC power supply and housed within a quartz tube case. The heater dimensions were 50 × 5 × 1.5 mm^3^, and a surface temperature of about 1500 °C within a local heating area of 5 × 10 mm^2^ could be achieved within about 10 s. For long-term testing, the source material (about 160 mg) was positioned at the highest temperature point. The quartz tube case was 70 mm in diameter and 140 mm long. Clean (dry and filtered) air was used as the carrier gas, and the gas flow maintained at 25.0 L/min (Re = 572, laminar flow regime) using a mass flow controller (MFC, AERA, FC-7810CD-4 V, Japan) [12]. In the current study, the exposure system consisted of two chambers; AgNP exposure and fresh air controls. The generator used 8.5 LPM (liters per minute) to generate and 16.5 LPM for the aerosol dilution system. The total volume flow of air in each chamber was 25 LPM controlled by the mass flow controller. The flow rate to each port in the nose-only chamber was approximately 1 LPM, similar to the recommended flow of 0.75 L/min by Pauluhn [13].

### Monitoring of inhalation chamber and analysis of AgNPs

In each chamber, the nanoparticle distribution with respect to size was measured directly in real-time using a differential mobility analyzing system (DMAS); combining a differential mobility analyzer (DMA-20, 4220, range 6–225 nm, HCT Co., Ltd. Korea) and condensation particle counter (CPC, 3775, size range 4 nm- 3 μm, TSI INC., Shoreview, MN). Nanoparticles from 6 to 225 nm were measured using sheath air at 15 L/min and polydispersed aerosol air at 1.5 L/min for DMAS and CPC, respectively. In addition, the mass concentration of silver nanoparticle was determined chemically by atomic absorption spectrophotometer (AAS, Perkin-Elmer 900 T, Waltham, MA, USA) after sampling on a mixed cellulose ester, (MCE) filter (size: 37 mm and pore size 0.45 μm, SKC, UK) at a flow rate of 1.0 L/min [14]. Three samples were taken from ports of the inhalation chamber each day during the 5-days of exposure. The target mass concentrations of the generated AgNPs in the chamber were 1 mg/m^3^.

### Transmission electron microscopy (TEM)

TEM sample for analysis was collected on TEM grid (200 mesh, Veco, Eerbeek, Holland) for 3 min by a nanocollector (HCT Co., Ltd., Icheon, Korea) in a chamber during the exposure period, and visualized under TEM (Hitachi 7100, Japan). The diameters of 400 randomly selected particles were measured at a magnification of 100,000, and the silver particles analyzed using an energy-dispersive x-ray analyzer (EDX-200, Horiba, Japan) at an accelerating voltage of 75 kV.

### Animals and exposure

Six-week-old male, specific-pathogen-free (SPF) Sprague-Dawley rats were purchased from Orient Bio (Korea) and acclimated for 2 weeks before starting the experiments. During the acclimation and experimental periods, the rats were housed in polycarbonate cages (5 rats per cage) in a room with controlled temperature (23 ± 2 °C) and humidity (55 ± 7%) with a 12-h light/dark cycle. The rats were fed a rodent diet (Harlan Teklab, Plaster International Co., Seoul) and filtered water ad libitum. The rats were adapted to the nose-only tubes for a week with daily tube placement for 2 h. The 8-week-old rats, weighing 253.84 ± 1.84 g (Mean ± SD) for the males were then divided into 4 groups: fresh air control for 1- (4 rats) and 5-day (4 rats), and AgNP for 1- (5 rats) and 5-day (5 rats) exposures (6 h/day). The animals were examined daily on weekdays for any evidence of exposure-related effects, including respiratory, dermal, behavioral, nasal, or genitourinary changes suggestive of irritation. The body weights were evaluated at the time of purchase, at the time of grouping, once a week during the inhalation exposure, and before necropsy (results are not shown). The rat experiments were approved by the Hanyang University Institutional Animal Care and Use Committee in South Korea (HY-IACUC-2017-0143A).

### Lung burden measurement

After 1 (6-h) or 5-day exposure, animals were immediately sacrificed by anesthetizing via intraperitoneal injection of anesthetic agent (Pentobarbital, EntobarVR, Hanlim Pharm Co. Ltd., Seoul, Korea) in a dose of 150 mg/kg body weight. The animals in the control group were sacrificed first and all the instruments for dissection were thoroughly washed with 70% ethyl alcohol in between the dissections. The thorax was opened by cutting up through the diaphragm and ribs. After isolation of lungs, lung lobes were carefully separated, each lobe was weighted and fixed in 10% neutral buffer formalin, and used for lung burden analysis. The weight of each wet lung lobe is presented in Table 2. The lung burden of AgNPs was determined from lung content of silver analyzed by ICP-MS (PerkinElmer NEXION 300S, Concord, ON, Canada) based on the NIOSH 7300 method [14]. Accordingly, the lung lobes were digested with 5 ml of concentrated nitric acid using a microwave digestion system (MARS 230/60, CEM, Matthews, NC). After wet digestion using a microwave, the concentrations of silver in the digested solution were analyzed using an ICP-MS and calculated using the calibration curve prepared with the silver standard solution. The background silver for the digestion procedure also validated by the same procedure with nitric acid only, where the background intensity was similar to the blank solution, 0.1% nitric acid.

### Estimation of particle number per acinus

Number of AgNP per acinus was calculated based on the acini number per lobe provided by earlier by Barré et al. [15]. According to Barré et al. [16], the total number of acini remains constant throughout postnatal rat lung development. To estimate AgNP number per acinus, the mass per lobe was divided by single AgNP mass (based on CMD 20 nm) to obtain total AgNP number per lobe, and the number of AgNP per lobe was divided by the number of acini per lobe to obtain the particle number per acinus.

1 $$ \frac{\mathrm{Ag}\ \mathrm{mass}/\mathrm{lobe}}{single\ AgNP\ mass}= AgNPs\  per\  lobe $$

2 $$ \frac{\mathrm{AgNPs}\ \mathrm{per}\ \mathrm{lobe}}{Number\ acini\  per\  lobe}= AgNP\ number\  per\  acinus $$

### Statistical analysis

All the results are expressed as the means ± standard deviation (SD). An analysis of variance (ANOVA) test and Duncan’s multiple range tests were used to compare the silver content among different lobes. Level of significance was set at *P* < 0.05 and *P* < 0.01.

## Results

### Silver nanoparticle distribution and mass concentration

**Fig. 1:**
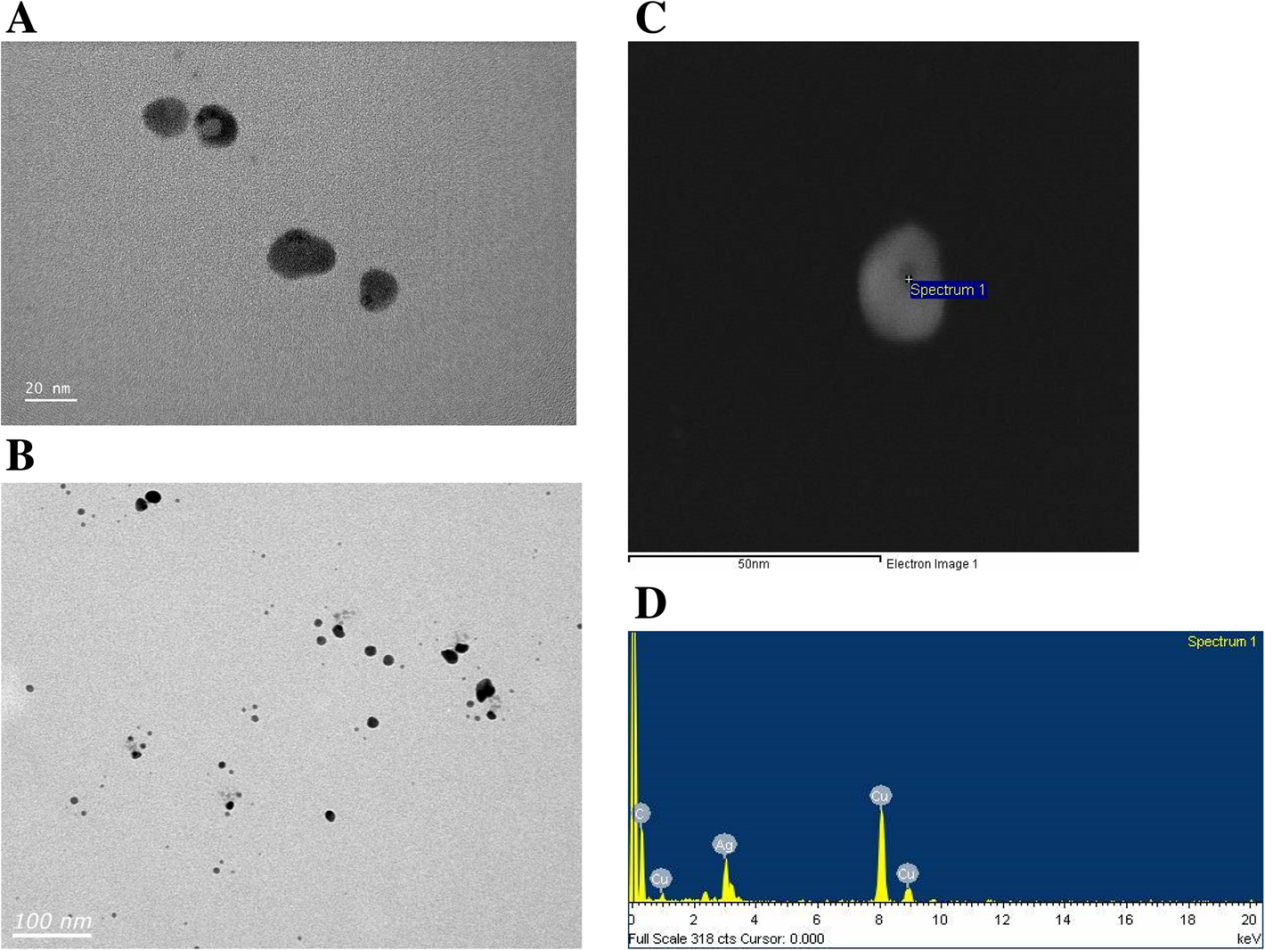
FE-TEM of result for AgNP sampled from a nose-only chamber. **a** Non-aggregated/agglomerated AgNP (scale 20 nm); **b** AgNP (scale 100 nm), **c** a particle process for EDX analysis (scale 50 nm) was marked as “spectrum1”; **d** the result of EDX indicates approximately 100% of silver

**Fig. 2:**
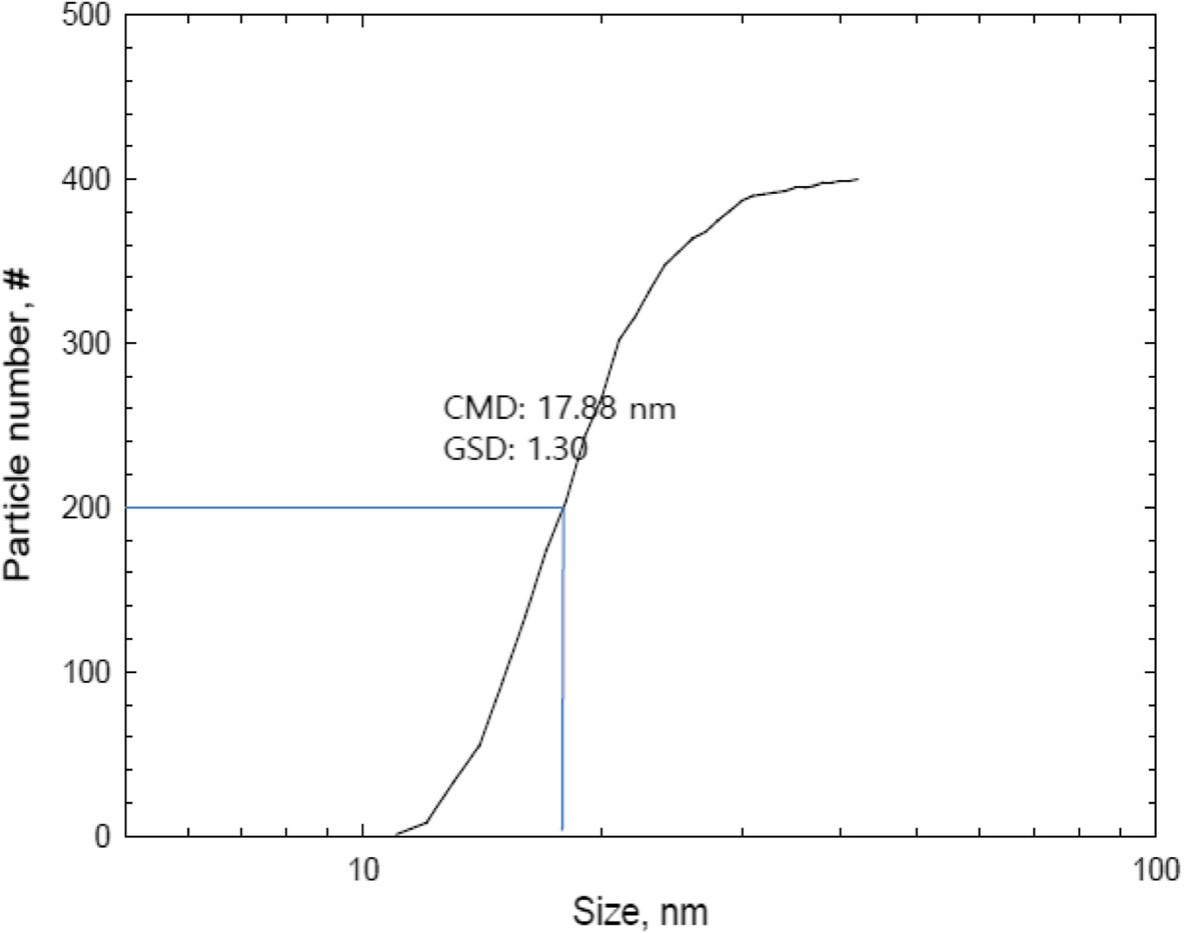
Size distribution of silver nanoparticles by TEM counting. Count median diameter (CMD), geometric standard deviation (GSD)

**Fig. 3:**
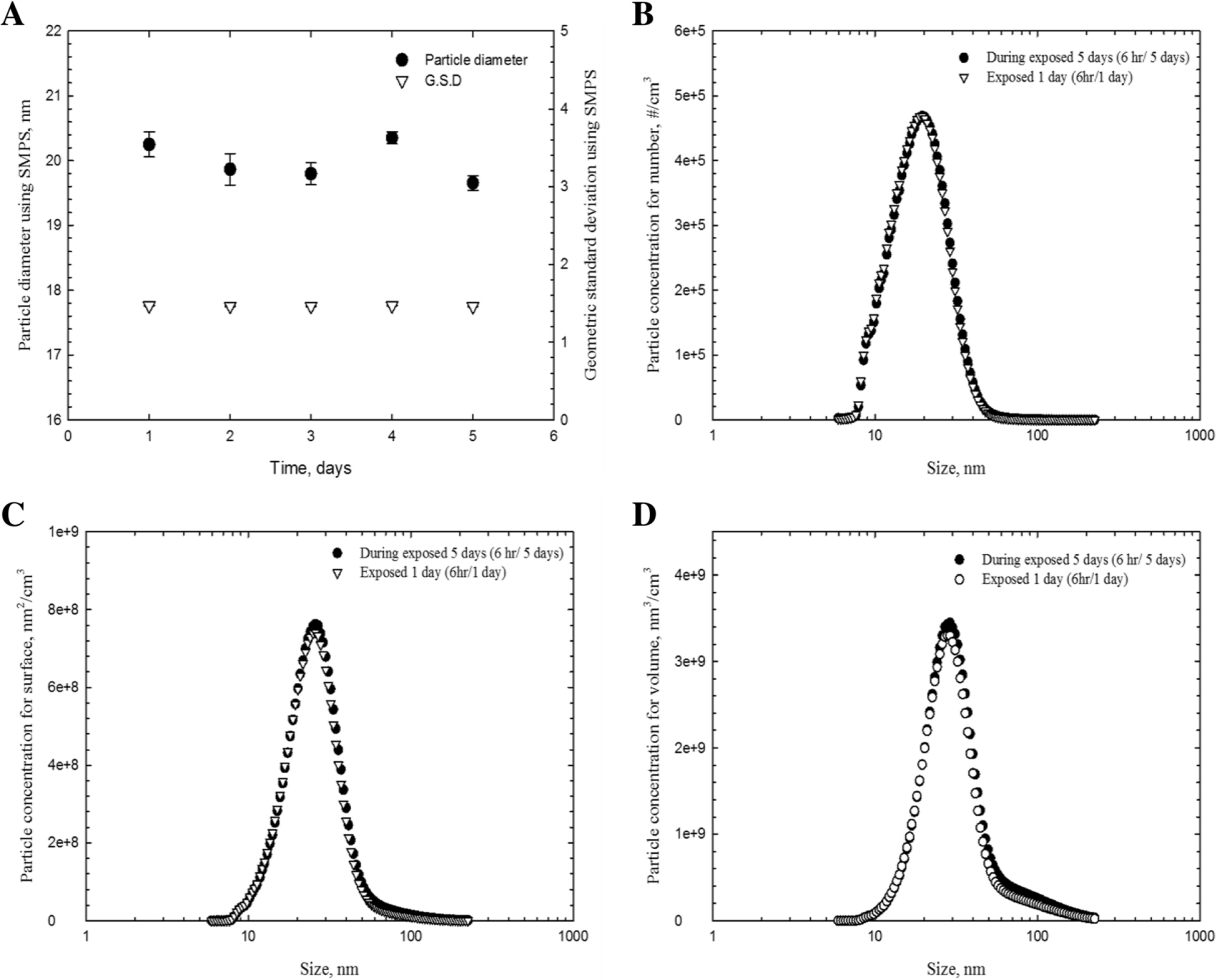
Particle diameter distribution of generated AgNP in exposure chamber during the 5-day exposure period by DMAS. **a** The particle diameter and GSD during the exposure period, **b** particle diameter distribution, **c** particle surface distribution and **d** particle volume distribution. These samples were measured during 6 h per day and 5-day per week

The total number concentration, CMD (count median diameter), GSD (geometric standard deviation) and surface area of AgNPs measured by the DMAS were 1.31 × 10^7^ particles/cm^3^, 19.98 nm, 1.46, and 1.91 × 10^10^ nm^2^/cm^3^, respectively (Table 1). Mass concentration estimated by DMAS was 923.28 ± 60.91 μg/m^3^ and mass concentration analyzed by AAS after filter sampling was 964.67 ± 65.35 μg/m^3^ (Table 1). The silver nanoparticles observed by TEM were spherical in shape and non-aggregated/agglomerated in form, with diameters under 42.4 nm (Fig. 1). The diameters were log-normally distributed between 11 and 42.4 nm, and the CMD and GSD were 17.88 nm and 1.3, respectively (Fig. 2). Figure 3 shows particle diameter distribution of generated AgNP in exposure chamber during the 5-day exposure period by DMAS.

**Table 1:** Distribution of silver nanoparticles (Mean ± S.D) in exposure system

Silver nanoparticles	Concentration
Number (particles/cm^3^) ^a^	1.31 × 10^7^ ± 2.57 × 10^5^
CMD^a^(nm) and GSD	19.98, 1.46
Surface area (nm^2^/cm^3^) ^a^	1.91 × 10^10^ ± 8.75 × 10^8^
Volume (nm^3^/cm^3^) ^a^	8.80 × 10^10^ ± 1.27 × 10^9^
Mass concentration (μg/m^3^) ^b^	923.28 ± 60.91 (DMAS); 964.67 ± 65.35 (AAS) ^b^
One particle mass (ng) ^b^	7.06 × 10^− 8^ ± 3.93 × 10^− 9^

^a^The particle concentration, surface area, volume and distribution were determined by DMAS; ^b^ The concentration was analyzed by AAS; *CMD* count median diameter, *GSD* Geometric standard deviation, *AAS* atomic absorption spectrometer

### Silver nanoparticle deposition/retention to the lung lobes after 1-day (6-h) exposure

Silver nanoparticle deposition/retention per lobe is shown in Table 2. Silver nanoparticles were retained to RCr (right cranial lobe), RM (right middle lobe), RCa (right caudal lobe), RA (right accessory lobe), and LL (left lung) for 10.35, 14.31, 27.1, 13.49%, and 34.75% of total lung deposits/retention, respectively, yet silver nanoparticles were evenly deposited/retained to the lung lobe in terms of μg/g of lung tissue (Table 2). When the deposition was expressed as a number of silver nanoparticle per acinus or deposition amount (ng/acinus), there was no statistically significant difference among lobes when comparing with other lobes. The AgNPs were evenly deposited/retained to the acinus in terms of mass and number (Table 2).

**Table 2:** Silver concentration in each a lung lobe (RCr, RM, RCa, and LL) at exposed 1-day (mean ± S.D) (*n* = 5)

Lobes	Deposition / Retention (μg/g)^a^	Lung weight (g)^b^ (% of total weight)	Retention (μg)/lobe^c^ (% of total)	Number of acini^d^ (range)	Retention (ng)/acinus^e^ (range)	No of AgNP (×10^6^)/acinus^f^ (range)
RCr	10.62 ± 1.80	0.10 ± 0.01 (11.1)	1.11 ± 0.23 (10.35%)	689 ± 138 (551–827)	1.61 ± 0.33 (1.28–1.94)	22.80 ± 4.65 (18.16–27.45)
RM	13.64 ± 1.68	0.11 ± 0.01 (12.2)	1.53 ± 0.26 (14.31%)	734 ± 66 (668–800)	2.09 ± 0.36 (1.73–2.45)	29.59 ± 5.06 (24.53–34.64)
RCa	11.00 ± 1.51	0.27 ± 0.03 (30)	2.91 ± 0.32 (27.10%)	1808 ± 103 (1705–1911)	1.61 ± 0.18 (1.43–1.79)	22.75 ± 2.54 (20.21–25.30)
RA	14.42 ± 1.48	0.10 ± 0.01 (11.1)	1.45 ± 0.20 (13.49%)	686 ± 89 (597–775)	2.11 ± 0.30 (1.81–2.40)	29.84 ± 4.19 (25.65–34.03)
RL^g^	12.02 ± 1.09	0.58 ± 0.03 (64.4)	7.00 ± 0.64 (65.25%)	3970 ± 329 (3641–4299)	1.76 ± 0.16 (1.60–1.92)	24.93 ± 2.29 (27.22–22.64)
LL	11.63 ± 1.25	0.32 ± 0.02 (35.6)	3.73 ± 0.26 (34.75%)	1973 ± 192 (1781–2165)	1.89 ± 0.13 (1.76–2.02)	26.73 ± 1.86 (24.87–28.59)
Total lung	11.88 ± 0.73	0.90 ± 0.05	10.72 ± 0.53	5943 ± 521 (5622–6264)	1.80 ± 0.09 (1.72–1.89)	25.54 ± 1.26 (24.28–26.80)

Statistics analysis was three point using ANOVA that deposition, deposition amount/acinus and No of AgNP/acinus; *RCr* right cranial, *RM* right median, *RCa* right caudal, *RA* right accessory lobe, *RL* right lung, *LL* left lung; ^a^ μg/L (ICP-MS; ppb) × 0.005 L (end of volume) / lobe weight = silver concentration in 1 Gram; ^b^ Each a lobe weight; ^c^ Retained mass concentration x lung lobe weight; ^d^ Acinus number of each a lung lobe [15]; ^e^ mass retained /g of lung lobe / number of acinus (ng); ^f^ Retained mass concentration of lobe / one particle mass of AgNP (DMAS particle concentration / DMAS particle mass) / number of acinus. Single particle mass was based on CMD 19.984 nm without considering range of particle size distribution and dissolution of AgNP; ^g^ whole right lung

### Silver nanoparticle retention to the lung lobes after the 5-day exposure

The retention pattern shown in 5-day exposure was similar to the pattern in 1-day (6-h) exposure showing no statistically significant difference among the lobes. There was also no statistically significant difference among the lobes in terms of retained amount or number of AgNP/acinus (Table 3). The percentages of retention per lobe were very similar to the 1-day exposure. Comparing the deposition/retention of 1-day exposure with the retention of 5-day exposure, 30% of silver was cleared in terms of total lung deposition during 5-day exposure. RCr, RM, RCa, RA, and LL cleared the silver nanoparticles 23.96, 27.06, 25.15, 32.55 and 36.19%, respectively. The left lung showed the highest clearance among the lobes (Table 4). When assuming that 1 day (6-h) data are all deposition, there is some uncertainty in a dissolution of AgNP during 6-h exposure.

**Table 3:** Silver concentration in each a lung lobe (RCr, RM, RCa, RA and LL) at exposed 5-day (mean ± S.D) (n = 5)

Lobes	Retention (μg/g)^a^	Lung weight (g)^b^ (% of total weight)	Retention (μg)/lobe^c^	Number of acini^d^ (range)	Retention (ng)/acinus^e^ (range)	No of AgNP (×10^6^)/acinus^f^ (range)
RCr	28.41 ± 3.88	0.15 ± 0.01 (11.1)	4.22 ± 0.84 (11.27%)	689 ± 138 (551–827)	6.13 ± 1.22 (4.91–7.35)	86.78 ± 17.22 (69.56–104.00)
RM	36.21 ± 2.68	0.15 ± 0.01 (12.2)	5.58 ± 0.60 (14.88%)	734 ± 66 (668–800)	7.59 ± 0.81 (6.78–8.41)	107.54 ± 11.53 (96.01–119.07)
RCa	31.18 ± 3.14	0.35 ± 0.01 (30)	10.89 ± 0.82 (29.06%)	1808 ± 103 (1705–1911)	6.02 ± 0.46 (5.57–6.48)	85.27 ± 6.42 (78.84–91.69)
RA	36.82 ± 5.01	0.13 ± 0.02 (11.1)	4.89 ± 0.50 (13.4%)	686 ± 89 (597–775)	7.13 ± 0.72 (6.40–7.85)	100.87 ± 10.27 (90.60–111.13)
RL^g^	32.59 ± 2.01	0.79 ± 0.04 (64.4)	25.58 ± 1.43 (68.26%)	3970 ± 329 (3641–4299)	6.44 ± 0.36 (6.08–6.80)	96.25 ± 5.06 (96.25–86.13)
LL	26.80 ± 7.68	0.45 ± 0.03 (35.6)	11.90 ± 3.02 (31.74%)	1973 ± 192 (1781–2165)	6.03 ± 1.53 (4.50–7.56)	85.35 ± 21.64 (63.72–106.99)
Total lung	30.49 ± 3.81	1.23 ± 0.07	37.47 ± 3.51	5943 ± 521 (5622–6264)	6.30 ± 0.59 (5.72–6.89)	89.26 ± 8.36 (80.90–97.62)

Statistics analysis was three point using ANOVA that deposition, deposition amount/acinus and No of AgNP/acinus; *RCr* right cranial, *RM* right median, *RCa* right caudal, *RA* right accessory lobe, *RL* right lung, *LL* left lung; ^a^ μg/L (ICP-MS; ppb) × 0.005 L (end of volume) / lobe weight = silver concentration in 1 Gram; ^b^ Each a lobe weight; ^c^ Retained mass concentration x lung lobe weight; ^d^ Acinus number of each a lung lobe [15]; ^e^ mass retained /g of lung lobe / number of acinus (ng); ^f^ Retained mass concentration of lobe / one particle mass of AgNP (DMAS particle concentration / DMAS particle mass) / number of acinus. Single particle mass was based on CMD 19.984 nm without considering range of particle size distribution and dissolution of AgNP; ^g^ whole right lung

**Table 4:** Percent of clearance during 5-day exposure

Lobes	1-day measured	5-day accumulated without clearance	5-day measured	Estimated clearance
	Deposition (μg)/lobe	(μg)/lobe	(μg)/lobe	%
RCr	1.11	5.55	4.22	23.96
RM	1.53	7.65	5.58	27.06
RCa	2.91	14.55	10.89	25.15
RA	1.45	7.25	4.89	32.55
RL	7.00	35.00	25.58	26.91
LL	3.73	18.65	11.90	36.19
Total lung	10.72	53.60	37.47	30.09

*RCr* right cranial, *RM* right median, *RCa* right caudal, *RA* right accessory lobe, *RL* whole right lung, *LL* left lung; Estimated clearance was calculated by (5-day accumulated without clearance – 5 day measured) / (5-day accumulated without clearance) × 100

## Discussion

In this investigation, the AgNPs with an average diameter of 20 nm were inhaled by experimental animals to maximize deposition to the pulmonary region. The evenness of deposition to lung lobes was examined with their mass concentration in each lobe. Results have shown that there was no statistically significant difference in AgNPs deposition right after 6-h exposure among the lobes in terms of silver mass/g of lung tissue, silver mass/acinus or silver nanoparticle number/acinus. Quantitative lung burden measurement leading to clearance kinetics and T_1/2_ uses the total mass of retained particle per lung calculated from the mass concentration per gram of lung tissue. Current OECD 412 and 413 mandate use of the right lung for lung burden measurement and the left lung for histopathology. Our result showed no statistically significant difference in mass concentration per gram of lung tissue among the right lung lobes as well as comparing with the left lobe.

Inhalation studies are expensive and time-consuming. Especially the revised OECD subacute and subchronic inhalation toxicity test guideline 412 and 413 require additional efforts and animal number to analyze BALF and lung burden measurement after exposure and during post-exposure observation [3, 4]. This, in turn, will mean that the required animal number will be doubled unless both lavage and lung burden can be done in the same animals; if mandatory and optional lung burden measurements were conducted. This is for the fact that according to the guidelines, the BALF assay and lung burden measurement should be performed in the right lung and the left lung to be used for histopathological evaluation.

The results obtained from our study indicated that nanoparticle deposited evenly in all of the lung lobes in terms of mass concentration μg silver per gram of lung tissue, μg silver per acinus, and number concentration per acinus. As the rat right lung has 4 lobes; RCr, RM, RCa, and RA, it is suggested that one lobe may be used for lung burden measurement, and the remaining lobes may be used for BALF collection after appropriate occlusion of the lobe for lung burden measurement. This, in turn, will result in a reduced number of animals used leading to also a reduction in experimental cost.

The total number of acini remains constant throughout postnatal rat lung development. Lung volume is achieved by an increase of the acinar volume and not by an increase of acinar number [15, 16]. The main portion of the lung lobe is acini, which are the major particle deposition site in the lung [17]. Therefore, estimating particle deposition to the lung lobes based on the retained mass per acinus or particle number per acinus should be similar to expressing retained amount per gram of lung or lobar tissue which is most convincing to describe evenness of deposition because it can be measured.

Several previous studies on the deposition of particles to the lung lobes using rat or hamster indicated that particles were preferentially deposited in the apical regions of the right lung. When the pulmonary deposition was tested with particles ranging from of 0.2–3.05 μm, the apical lobe of the right lung received the largest percentage deposition when comparing with its percentage of total lung weight (the left lung was not separated into upper and lower region) [18]. In our study, percent retained per lobe and percent weight per lobe are very similar, almost the same as seen Tables 2 and 3. A particle deposition study using mono-dispersed (MMAD 1.5 μm) and poly-dispersed particles (MMAD 1.9 μm) also showed the right apical lobes consistently contained more activity per unit weight [19]. In our study, percent retained per lobe and percent weight per lobe are very similar, almost the same as seen Tables 2 and 3. Another particle deposition study in hamsters labeled with ^99m^TC (0.01–3.0 μm; 10% ≤0.1 μm; 70% 0.1–0.4 μm, and 4% ≥1.0 μm) resulted in more even distribution with preferential deposition in the apical lobes [20]. When particle deposition fraction in the nasal passages and in various lobes and regions of the Long-Evans rat lung was measured following a nose-only exposure to ^59^Fe radiolabeled monodisperse condensation particles of triphenylphosphate particles of 11 sizes ranging from 0.9–4.2 μm, the left lung received higher deposition than each lobe of the right lung. In the right lung, the caudal and accessory lobes had the highest and lowest deposition, respectively. Depositions in the right medial and right cranial lobes were similar [21]. The differences between our study to other previous studies are particle size and associated deposition mechanisms. We used a well dispersed 20 nm AgNP aerosol which is deposited in the respiratory tract almost exclusively by diffusion, while other studies using submicron to micron size particles could be operated with different deposition mechanisms such as impaction and sedimentation. Therefore, diffusion dominant deposition resulted in even particle deposition among the lung lobes, indicating a significant difference in terms of evenness of lung deposition between nano-sized and micron-sized aerosols. Thus, caution should be exercised when using a single lung lobe to measure lung burden and clearance after exposure to larger particles having impaction and sedimentation as dominant deposition mechanisms.

Our results show that the estimated clearance from the left lung is higher than the right lung. Clearance of deposited particles from the conducting airway and alveolar clearance processes involves several factors including the pulmonary macrophage, mucociliary clearance, dissolution, transport to the systemic circulation, and translocation via regional lymphatic vessels [22]. At this stage, it is difficult to define what may be the main reason for the observed difference in clearance between the right lung and the left lung. This difference might be explained by the different branching patterns of these lobes. The left lung is highly asymmetric, while the branching pattern of the right lung lobes is more symmetric. So, mucociliary clearance from the large longitudinal airway in the left lung of small rodents might be responsible for this difference. Furthermore, it should be noted that our findings of evenness of inhaled nanoparticle deposition and retention in the lung lobes are based on the ~ 20 nm size of the AgNP and consider also the male Sprague-Dawley rat strain used.

The use of MPPD modeling to estimate the retention of AgNPs in this study at the end of 1 or 5 days of exposure may not be advisable because of potential clearance by dissolution during the 6 h exposure period. This deduction was based on the observation that when using the variables presented in Table 1, the MPPD (version 3.04) estimated 27% of pulmonary deposition (Additional file 1) after 6-h (1-day) exposure, while the actual deposition fraction measured by silver mass weight analysis was 13.4%. Although deposition modeling using MPPD is an appropriate deposition model for all inhaled particles, when applied to results of several hours of exposure it may not predict the retained particle load of high dissolution nanoparticles such as silver, zinc oxide, and copper oxide because significant clearance has occurred, unless their in vivo dissolution rate is known and can be applied. The dissolution rate of the phagocytized particles inside a phagolysosome is an important determinant of the clearance [23]. Without knowing the actual dissolution rate of AgNPs in the rat lung, our study design included the measurement of the actually retained Ag in the lung, making it unnecessary to use predictive MPPD modeling. Low dissolution particles such as TiO_2_, crystalline silica, or gold nanoparticles would be correctly predicted by MPPD because within 6 h the loss due to alveolar macrophage clearance is only 0.25% of lung burden in rats [24]. Predicted by MPPD modeling, the AgNPs with average 20 nm have a maximum deposition in the pulmonary region [5–8], but the dissolution half-times of silver nanoparticles may range from 5.4 s to 30 days depending on experimental condition and nanoparticle size [25–28]. Principally, in vivo particle dissolution half-times can be estimated from the accumulation kinetics of a repeat inhalation study [29], but our study was not designed to do this. It is conceivable that rapid phagolysosomwal dissolution by macrophages may remove a significant amount of deposited AgNPS within 6 h. Our previous autometallographic study on the lung tissue after 90-day AgNP exposure clearly indicated that AgNPs were ingested by alveolar macrophages (Additional file 2) [30].

In order to investigate and confirm the role of dissolution as well as the role of fibrous form on the lung burden and clearance of poorly soluble nanomaterials, further studies are presently being conducted in our laboratories with gold nanoparticles with low dissolution rate as well as with carbon nanotubes with fibrous form to assess their deposition and retention pattern in the different lobes of the rat lung.

## Conclusions

This study evaluates the variability in nanoparticle deposition to specific lung lobes of rats following inhalation, and as such is highly relevant for determining if and when the particle dose differs in specific lung lobes or if all lung lobes receive the same burden. It could be shown that 20 nm inhaled well-dispersed AgNPs deposited and were retained evenly in all lung lobes in terms of Ag mass per gram of lung tissue, Ag mass per acinus and Ag number per acinus after exposure of rats for 1 day or 5 days. The evenness of deposition of inhaled NPs per unit weight of rat lung lobes is a novel finding which contrasts earlier studies with inhaled micron-sized particles. These findings indicate that any lobe in the lung may be used to determine total lung burden as long as the same lobe is sampled for these measurements. The remaining lobes of the lung may then be used for BALF and histopathological analysis provided proper occlusion of the lung lobe is performed for lung burden measurement. For longer-term inhalation studies with poorly soluble NPs, it would still be necessary to determine the evenness of lobar retention under those conditions. This could be different in long-term studies where clearance comes in to play, which may be different for different lung lobes. Our findings may, however, be limited to the particle size distribution, test material and strain of animals used in this study.

## Additional files

Additional file 1: MPPD estimation of AgNP deposition to the lung region after 6-hr (1-day) exposure. TB, tracheobronchial; P, pulmonary. (DOCX 47 kb)

Additional file 2: Lung section stained using autometallography (From Miller et al., 2016). (DOCX 307 kb)
