# Supplementary material for: Lobar evenness of deposition/retention in rat lungs of inhaled silver nanoparticles: an approach for reducing animal use while maximizing endpoints
Source: Part Fibre Toxicol. 2019 Jan 7;16:2. doi: 10.1186/s12989-018-0286-9 (PMC6322301; doi:10.1186/s12989-018-0286-9)

Additional file 1. MPPD estimation of AgNP deposition to the lung region after 6-hr (1-day) exposure. TB, tracheobronchial; P, pulmonary.


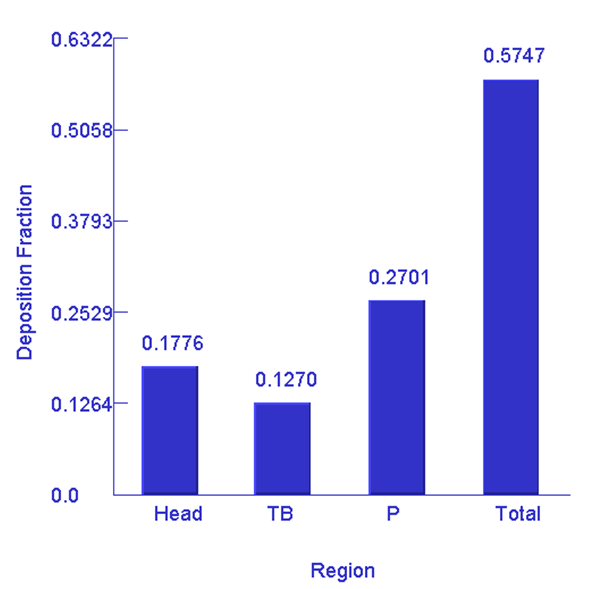

Supplement: Supplementary file 1 — MPPD estimation of AgNP deposition to the lung region after 6-hr (1-day) exposure. TB, tracheobronchial; P, pulmonary. (DOCX 47 kb) [file 12989_2018_286_MOESM1_ESM.docx]
