# Supplementary material for: Lobar evenness of deposition/retention in rat lungs of inhaled silver nanoparticles: an approach for reducing animal use while maximizing endpoints
Source: Part Fibre Toxicol. 2019 Jan 7;16:2. doi: 10.1186/s12989-018-0286-9 (PMC6322301; doi:10.1186/s12989-018-0286-9)
Supplement: Supplementary file 2 — Lung section stained using autometallography (From Miller et al., 2016). (DOCX 307 kb) [file 12989_2018_286_MOESM2_ESM.docx]

Additional file 2. Lung section stained using autometallography (From Miller et al., 2015).
